# The Effects of Balance Training on Static and Dynamic Postural Stability Indices After Acute ACL Reconstruction

**DOI:** 10.5539/gjhs.v8n4p68

**Published:** 2015-07-30

**Authors:** Asghar Akbari, Fateme Ghiasi, Mohsen Mir, Mohammad Hosseinifar

**Affiliations:** 1 Health Promotion Research Center, Zahedan University of Medical Sciences, Zahedan, Iran; 2 Zahedan University of Medical Sciences, Zahedan, Iran

**Keywords:** Anterior Cruciate Ligament (ACL) Reconstruction, static stability indices, dynamic stability indices

## Abstract

**Background:**

Proprioception and postural stability play an important role in knee movements. However, there are controversies about the overall recovery time of proprioception following knee surgery and onset of balance and neuromuscular training after ACL reconstruction. Therefore, it is necessary to evaluate the effect of balance training in early stage of knee rehabilitation after anterior cruciate ligament (ACL) reconstruction. The purpose of this study was to evaluate the effect of balance exercises on postural stability indices in subjects with anterior cruciate ligament (ACL) reconstruction.

**Methods:**

The study was a controlled randomized trial study. Twenty four patients who had ACL reconstructed (balance training group) and twenty four healthy adults without any knee injury (control group) were recruited in the study. The balance exercises group performed balance exercises for 2 weeks. Before and after the interventions, overall, anteroposterior, and mediolateral stability indices were measured with a Biodex Balance System in bilateral and unilateral stance positions with the eyes open and closed. T-tests were used for statistical analysis (p<0.05).

**Results:**

Results showed that amount of static stability indices did not change after training and there were not significant differences in static stability indices before and after balance training (p>0.05). Although amount of dynamic stability indices decreased, there were not significant differences in dynamic stability indices before and after balance training (p>0.05). Amount of dynamic stability indices were decreased in balance training group, however, there were not significant differences between groups (p>0.05).

**Conclusion:**

These results support that balance exercise could partially improved dynamic stability indices in early stage of ACL reconstruction rehabilitation. The results of this study suggest that balance exercises should be part of the rehabilitation program following ACL reconstruction.

## 1. Introduction

Anterior cruciate ligament injury is the most common knee ligament injury (Risberg et al., 2007; Snyder-Mackler et al., 1997). Deficient in neuromuscular control of the lower extremity is one of the main impairments following ACL injury. Therefore, there are several rehabilitation protocols after the injury for retraining of the knee neuromuscular control (Risberg et al., 2007; Chmielewski et al., 2002; Lewek et al., 2002; Risberg et al., 2004). The re-establishment of neuromuscular control of the lower extremity has recently been recognized as one of the keys to restoring dynamic joint stability and functional movement patterns (Risberg et al., 2007; Chmielewski et al., 2002; Rudolph et al., 1998). On the other hand, there are evidences that injury of the anterior cruciate ligament (ACL) and ACL surgical reconstruction may lead to proprioceptive deficits (Cooper et al., 2005; Barrack et al., 1989; Corrigan et al., 1992; Barrett, 1991). Therefore, improvement of the neuromuscular control of the knee following ACL injury or reconstruction makes possible better outcomes to return the functional activities and to reduce the rate of re-injury (Cooper et al., 2005).

A theoretical foundation exists that proprioceptive and balance training may improve the nervous system's ability to synchronize muscular activity around a joint to improve dynamic knee joint stability, and to improve outcomes in people following injury or surgery to the ACL. Cooper et al. (2005) in systematic review confirm that proprioceptive and balance exercise may improve outcomes in people with ACL deficiency, and that some modest benefits are apparent for people who have undergone ACL reconstruction (Cooper et al., 2005). Johansson et al. (1991) suggested that stimulation of mechanoreceptors in joint structures stimulate the sensitivity of muscle spindles around the joint and create a state of awareness of muscles to respond to forces applied to the joint and in that way, improve active joint stability. Although ACL injuries disrupt local mechanoreceptors, compensatory activation of other knee joint mechanoreceptors create compensatory muscle activation to promote the joint stabilization (Johansson et al., 1991). These compensatory neuromuscular patterns may be developed and enhanced by utilizing neuromuscular and proprioceptive protocols to improve postural stability and functional activities. For these reasons, balance and proprioceptive exercises defined as ACL rehabilitation protocols and these exercises have been generally used in the clinical setting (Cooper et al., 2005; Brukner & Khan, 2001; Swanik et al., 1997). Finally, it seems that Proprioceptive and balance exercises appear to be a safe form of rehabilitation exercises after ACL reconstruction (Cooper et al., 2005).

However, it should be noted that cooper et al. (2005) said that these findings are based on a limited amount of research, from five papers, only four papers investigated rehabilitation for people with ACL deficiency and one paper investigating rehabilitation following ACL reconstruction (Ageberg et al., 2001; Ageberg, 2002; Beard et al., 1994; Fitzgerald et al., 2000; Liu-Lui-Ambrose et al., 2003; Zatterstrom et al., 2000). The results are consistent of evidence for ACL rehabilitation conducted by Risberg et al. (2004), which included a brief evaluation of neuromuscular training for ACL rehabilitation based on three of the studies included in this review (Cooper et al., 2005; Liu-Ambrose et al., 2003; Beard et al., 1994; Fitzgerald et al., 2000). Also, there is no study to report direct effect of balance training exercises to improvement postural stability after ACL reconstruction in early stage of rehabilitation program. Therefore, it was the aim of the current study to evaluate effect of balance program in postural stability during the early phase of rehabilitation after ACL reconstructive surgery. It was hypothesized that a balance program would lead to improve static and dynamic postural stability after ACL reconstructive surgery.

## 2. Methods

### 2.1 Subjects

Twenty four men with ACL reconstruction were recruited among of thirty seven subjects who referred for rehabilitation services to the physiotherapy Center of Zahedan University of Medical Science. Participants were selected by simple non-probability sampling method. Also, twenty four healthy male students were selected through simple non-probability sampling method.

All protocols were approved by the ethical committee of Zahedan university of Medical Sciences, and all participants signed written informed consents.

#### 2.1.1 Inclusion and Exclusion Criteria

Subjects with ACL reconstruction, four weeks after surgery, men, 16-35 yrs., no pain in the ankle joint; not being involved in any sports activity in the period of this study; healthy sensory motor function in the lower limbs; no history of neuromuscular disease, vertigo or any uncorrected visual problems, any kind of knee injury or lower limb surgery, taking sedative medication, cardiovascular, neurologic, or pulmonary disease, balance problems, rheumatoid disease, fracture and contracture, osteoporosis, osteoarthritis, and psychological disease; and a body mass index of between 17 and 25, and ability of balance in single leg position were included in this study (Akbari et al., 2014a, 2014b; Cooper et al., 2005).

In control group, subjects were tested for the health of the muscle and ligaments around the ankle joint by anterior and posterior drawer tests and joint glides (Akbari et al., 2014a, 2014b).

Subjects were excluded if they currently enrolled in physical therapy or other training program and or did not complete sessions of treatment in the current study (Akbari et al., 2014a, 2014b).

### 2.2 Sample Size

The sample size was determined based on a pilot study. Ten subjects were divided randomly into two equal groups, and the main part of study was conducted on them. The means and SDs for the parameters from this pilot study, with α= 0.05 and 90% power were used to calculate the sample size.

### 2.3 Procedures

The overall stability index (OSI), anteroposterior stability index (APSI), and mediolateral stability index (MLSI) were recorded with a Biodex Balance System (SD 950-340, Biodex Medical Systems, Inc., Shirley, NY, USA).

The Biodex system has a circular deck with a 55 cm diameter located 20 cm above the floor inside its body, which is able to tilt 20 degrees from the horizontal position to all sides. The overall stability index shows the variance in plate deviation from the horizontal plane. The anteroposterior and mediolateral indices show the deviation of the plate from the horizontal position in the sagittal and frontal planes, respectively. The scores for the indices show the level of deviation from the horizontal position, so the lower scores indicate better balance (Akbari et al., 2014a, 2014b, Manual of Biodex System).

The subjects stood on the balance board without shoes and stockings. The right heel was placed at the intersection of the lines from E and 9. The left heel was placed on the intersection of the lines from F and 12. The feet were 20 degrees out toed. The hands were laid one across the other on the thorax. The difficulty level of the test in the double leg standing position with the eyes open and closed was decreased from level 6 to level 3. In other words, the difficulty level in a 20-second trial changed from six to three. The trial began with level six and finished in level three. In the single leg standing positions with the eyes closed and open, the difficulty level changed from eight to five gradually in accordance with previous method (Akbari et al., 2014a, 2014b; Manual of Biodex System).

The subjects became familiar with the testing protocol in a pretesting session. Dynamic postural stability test was performed in the double leg standing position with the eyes open and closed and in the single leg standing position with the eyes open and closed on the right and left legs. Each test included three trials that lasted 20 seconds with a 10-second interval between trials for rest. A mean score was calculated from the three test evaluations. There was five-minute rest period between tests.

### 2.4 Interventions

In balance training group, subjects were trained with balance training rehabilitation program for 30 minutes, 6 days in week, for 12 sessions. Exercises were including: Single leg stance with eyes closed and opened, Step-up exercises: anterior, lateral, posterior for uninvolved and involved leg (Risberg et al., 2007). After completing the sessions of treatment, all of assessments were measured again.

### 2.5 Statistical Analysis

Results were presented as mean values and standard deviation (SD). Criterion of significancy was set as p<0.05. Data analysis was performed with SPSS version 17. The assumption of a normal distribution was assessed using the K-S test. The assumption of equality of variances was tested using Levene's test. Paired t-test was used to compare balance variables before and after intervention. Independent t-test was used to compare differences between two groups after intervention.

## 3. Results

Twenty four patients with ACL reconstruction and twenty four healthy subjects were enrolled to this study between October 2012 and August 2013. Subjects were assigned in Healthy (Control) group or Balance training group. Table-1 provides demographic characteristics of both groups. There were no statistical differences in these variables between both groups. Any subjects were not dropped out in both groups.

**Table 1:** Demographic and baseline characteristics of subjects

	Control Group	Balance Training Group	P-Values
**Age, y**	20.33±10.9	22.33±11.03	0.65
**Weight, kg**	68.42±13.34	71.83±11.82	0.51
**Height, cm**	167.42±9.55	168.42±8.60	0.79
**BMI, kg/cm^2^**	21.95±3.38	20.73±2.45	0.15

Paired t-test was used to compare stability indices variables before and after intervention in balance training group. Table 2 has shown that amount of static stability indices did not change after training and there were not significant differences in static stability indices before and after balance training (p>0.05).

**Table 2:** Mean±SD static stability indices before and after balance training

	Before Balance Training (Mean±SD)	After Balance Training (Mean±SD)	p-value
**SMOSIBEO**	0.37±0.16	0.39±0.26	0.76
**SMOSIBEC**	1.47±0.7	1.28±0.62	0.15
**SMAPSIBEO**	0.28±0.14	0.32±0.21	0.53
**SMAPSIBEC**	1.32±0.61	0.99±0.53	0.09
**SMMLSIBEO**	0.16±0.07	0.17±0.14	0.73
**SMMLSIBEC**	0.44±0.32	0.52±0.43	0.56
**SMOSIUSIEO**	1.08±0.6	1.03±0.64	0.84
**SMOSIUSIEC**	3.95±1.91	3.02±1.08	0.16
**SMAPSIUSIEO**	0.76±0.51	0.73±0.5	0.89
**SMAPSIUSIEC**	3.44±2.05	2.58±1.72	0.24
**SMMLSIUSIEO**	0.62±0.25	0.59±0.26	0.81
**SMMLSIUSIEC**	1.34±0.38	1.16±0.34	0.91
**SMOSIUSUEO**	1.05±0.53	1.05±0.65	1
**SMOSIUSUEC**	3.3±1.45	2.45±0.63	0.69
**SMAPSIUSUEO**	0.6±0.13	0.85±0.16	0.26
**SMAPSIUSUEC**	2.63±1.39	2.63±1.39	0.07
**SMMLSIUSUEO**	0.48±0.22	0.45±0.23	0.74
**SMMLSIUSUEC**	1.36±0.49	1.23±0.42	0.4

* Significant P<0.05.

SMOSIBEO=STATIC MEAN OVERALL STABILITY INDEX BILATERAL STANCE EYES OPEN

SMOSIBEC=STATIC MEAN OVERALL STABILITY INDEX BILATERAL STANCE EYES CLOSE

SAPOSIBEO=STATIC MEAN ANTRO POSTERIOR STABILITY INDEX BILATERAL STANCE EYES OPEN

SAPOSIBEC=STATIC MEAN ANTRO POSTERIOR STABILITY INDEX BILATERAL STANCE EYES CLOSE

SMMLOSIBEO=STATIC MEAN MEDIO LATERAL STABILITY INDEX BILATERAL STANCE EYES OPEN

SMMLOSIBEC=STATIC MEAN MEDIO LATERAL STABILITY INDEX BILATERAL STANCE EYES CLOSE

SMOSIUSIEO=STATIC MEAN OVERALL STABILITY INDEX UNILATERAL INVOLVED STANCE EYES OPEN

SMOSIUSIEC=STATIC MEAN OVERALL STABILITY INDEX UNILATERAL INVOLVED STANCE EYES CLOSE

SAPOSIUSIEO=STATIC MEAN ANTRO POSTERIOR STABILITY INDEX UNILATERAL INVOLVED STANCE EYES OPEN

SAPOSIUSIEC=STATIC MEAN ANTRO POSTERIOR STABILITY INDEX UNILATERAL INVOLVED STANCE EYES CLOSE

SMLOSIUSIEO=STATIC MEAN MEDIO LATERAL STABILITY INDEX UNILATERAL INVOLVED STANCE EYES OPEN

SMLOSIUSIEC=STATIC MEAN MEDIO LATERAL STABILITY INDEX UNILATERAL INVOLVED STANCE EYES CLOSE

SMOSIUSUEO=STATIC MEAN OVERALL STABILITY INDEX UNILATERAL UNINVOLVED STANCE EYES OPEN

SMOSIUSUEC=STATIC MEAN OVERALL STABILITY INDEX UNILATERAL UN INVOLVED STANCE EYES CLOSE

SAPOSIUSUEO=STATIC MEAN ANTRO POSTERIOR STABILITY INDEX UNILATERALUN INVOLVED STANCE EYES OPEN

SAPOSIUSUEC=STATIC MEAN ANTRO POSTERIOR STABILITY INDEX UNILATERAL UNINVOLVED STANCE EYES CLOSE

SMLOSIUSUEO=STATIC MEAN MEDIO LATERAL STABILITY INDEX UNILATERALUN INVOLVED STANCE EYES OPEN

SMLOSIUSUEC=STATIC MEAN MEDIO LATERAL STABILITY INDEX UNILATERAL UNINVOLVED STANCE EYES CLOSE

Table 3 has shown that although amount of dynamic stability indices decreased, there were not significant differences in dynamic stability indices before and after balance training (p>0.05).

**Table 3:** Mean±SD Dynamic stability indices before and after balance training

	Before Balance Training (Mean±SD)	After Balance Training (Mean±SD)	p-value
**DMOSIBEO**	1.76±0.79	1.67±0.77	0.73
**DMOSIBEC**	6.5±2.56	5.16±3.16	0.4
**DMAPSIBEO**	1.38±0.5	1.12±0.58	0.38
**DMAPSIBEC**	5.12±2.22	4.03±2.58	0.43
**DMMLSIBEO**	0.95±0.51	0.96±0.41	0.95
**DMMLSIBEC**	2.99±0.25	2.53±1.44	0.52
**DMOSIUSIEO**	2.91±1.17	1.87±0.74	0.69
**DMOSIUSIEC**	5.8±1.58	4.06±1.9	0.1
**DMAPSIUSIEO**	2.05±0.93	1.15±0.36	0.02*
**DMAPSIUSIEC**	4.67±1.39	2.94±1.69	0.67
**DMMLSIUSIEO**	1.72±0.79	1.19±0.67	0.2
**DMMLSIUSIEC**	2.57±1.08	2.07±0.86	0.36
**DMOSIUSUEO**	2.04±0.57	1.86±0.94	0.59
**DMOSIUSUEC**	5.17±1.27	3.87±1.49	0.42
**DMAPSIUSUEO**	1.27±0.33	1.16±0.63	0.5
**DMAPSIUSUEC**	4.07±1.42	3.07±1.33	0.12
**DMMLSIUSUEO**	1.26±0.57	1.11±0.73	0.55
**DMMLSIUSUEC**	2.34±0.65	1.74±0.55	0.13

* Significant P<0.05.

DMOSIBEO=DYNAMIC MEAN OVERALL STABILITY INDEX BILATERAL STANCE EYES OPEN

DMOSIBEC=DYNAMIC MEAN OVERALL STABILITY INDEX BILATERAL STANCE EYES CLOSE

DAPOSIBEO=DYNAMIC MEAN ANTRO POSTERIOR STABILITY INDEX BILATERAL STANCE EYES OPEN

DAPOSIBEC=DYNAMIC MEAN ANTRO POSTERIOR STABILITY INDEXBILATERAL STANCE EYES CLOSE

DMMLOSIBEO=DYNAMIC MEAN MEDIO LATERAL STABILITY INDEX BILATERAL STANCE EYES OPEN

DMMLOSIBEC=DYNAMIC MEAN MEDIO LATERAL STABILITY INDEX BILATERAL STANCE EYES CLOSE

DMOSIUSIEO=DYNAMIC MEAN OVERALL STABILITY INDEX UNILATERAL INVOLVED STANCE EYES OPEN

DMOSIUSIEC=DYNAMIC MEAN OVERALL STABILITY INDEX UNILATERAL INVOLVED STANCE EYES CLOSE

DAPOSIUSIEO=DYNAMIC MEAN ANTRO POSTERIOR STABILITY INDEX UNILATERAL INVOLVED STANCE EYES OPEN

DAPOSIUSIEC=DYNAMIC MEAN ANTRO POSTERIOR STABILITY INDEX UNILATERAL INVOLVED STANCE EYES CLOSE

DMLOSIUSIEO=DYNAMIC MEAN MEDIO LATERAL STABILITY INDEX UNILATERAL INVOLVED STANCE EYES OPEN

DMLOSIUSIEC=DYNAMIC MEAN MEDIO LATERAL STABILITY INDEX UNILATERAL INVOLVED STANCE EYES CLOSE

DMOSIUSUEO=DYNAMIC MEAN OVERALL STABILITY INDEX UNILATERAL UNINVOLVED STANCE EYES OPEN

DMOSIUSUEC=DYNAMIC MEAN OVERALL STABILITY INDEX UNILATERAL UN INVOLVED STANCE EYES CLOSE

DAPOSIUSUEO=DYNAMIC MEAN ANTRO POSTERIOR STABILITY INDEX UNILATERALUN INVOLVED STANCE EYES OPEN

DAPOSIUSUEC=DYNAMIC MEAN ANTRO POSTERIOR STABILITY INDEX UNILATERAL UNINVOLVED STANCE EYES CLOSE

DMLOSIUSUEO=DYNAMIC MEAN MEDIO LATERAL STABILITY INDEX UNILATERALUN INVOLVED STANCE EYES OPEN

DMLOSIUSUEC=DYNAMIC MEAN MEDIO LATERAL STABILITY INDEX UNILATERAL UNINVOLVED STANCE EYES

Tables 4 and 5 have shown that amount of static and dynamic stability indices did not change and there were not significant differences in static and dynamic stability indices in control group (p>0.05).

**Table 4:** Mean±SD static stability indices in control group

	First day (Mean±SD)	After fifty weeks (Mean±SD)	p-value
**SMOSIBEO**	0.46±0.23	0.46±0.23	0.99
**SMOSIBEC**	1.84±0.38	1.84±0.38	0.99
**SMAPSIBEO**	0.38±0.23	0.38±0.23	0.99
**SMAPSIBEC**	0.98±0.42	0.98±0.42	0.99
**SMMLSIBEO**	1.84±0.72	1.84±0.72	0.99
**SMMLSIBEC**	0.49±0.27	0.49±0.27	0.99
**SMOSIUSIEO**	1.15±0.51	1.25±0.46	0.34
**SMOSIUSIEC**	2.92±1.38	2.83±1.52	0.34
**SMAPSIUSIEO**	0.82±0.4	0.9±0.38	0.34
**SMAPSIUSIEC**	2.2±1.2	2.1±1.33	0.34
**SMMLSIUSIEO**	0.6±0.39	0.8±0.62	0.34
**SMMLSIUSIEC**	1.59±0.87	1.45±0.87	0.34
**SMOSIUSUEO**	1.09±0.84	1.18±0.83	0.41
**SMOSIUSUEC**	2.93±1.16	2.83±1.33	0.34
**SMAPSIUSUEO**	0.75±0.52	0.83±0.54	0.34
**SMAPSIUSUEC**	2.26±1.11	2.33±1.07	0.34
**SMMLSIUSUEO**	0.62±0.67	0.62±0.67	0.99
**SMMLSIUSUEC**	1.35±0.37	1.35±0.37	0.99

* Significant P<0.05.

SMOSIBEO=STATIC MEAN OVERALL STABILITY INDEX BILATERAL STANCE EYES OPEN

SMOSIBEC=STATIC MEAN OVERALL STABILITY INDEX BILATERAL STANCE EYES CLOSE

SAPOSIBEO=STATIC MEAN ANTRO POSTERIOR STABILITY INDEX BILATERAL STANCE EYES OPEN

SAPOSIBEC=STATIC MEAN ANTRO POSTERIOR STABILITY INDEX BILATERAL STANCE EYES CLOSE

SMMLOSIBEO=STATIC MEAN MEDIO LATERAL STABILITY INDEX BILATERAL STANCE EYES OPEN

SMMLOSIBEC=STATIC MEAN MEDIO LATERAL STABILITY INDEX BILATERAL STANCE EYES CLOSE

SMOSIUSIEO=STATIC MEAN OVERALL STABILITY INDEX UNILATERAL INVOLVED STANCE EYES OPEN

SMOSIUSIEC=STATIC MEAN OVERALL STABILITY INDEX UNILATERAL INVOLVED STANCE EYES CLOSE

SAPOSIUSIEO=STATIC MEAN ANTRO POSTERIOR STABILITY INDEX UNILATERAL INVOLVED STANCE EYES OPEN

SAPOSIUSIEC=STATIC MEAN ANTRO POSTERIOR STABILITY INDEX UNILATERAL INVOLVED STANCE EYES CLOSE

SMLOSIUSIEO=STATIC MEAN MEDIO LATERAL STABILITY INDEX UNILATERAL INVOLVED STANCE EYES OPEN

SMLOSIUSIEC=STATIC MEAN MEDIO LATERAL STABILITY INDEX UNILATERAL INVOLVED STANCE EYES CLOSE

SMOSIUSUEO=STATIC MEAN OVERALL STABILITY INDEX UNILATERAL UNINVOLVED STANCE EYES OPEN

SMOSIUSUEC=STATIC MEAN OVERALL STABILITY INDEX UNILATERAL UN INVOLVED STANCE EYES CLOSE

SAPOSIUSUEO=STATIC MEAN ANTRO POSTERIOR STABILITY INDEX UNILATERALUN INVOLVED STANCE EYES OPEN

SAPOSIUSUEC=STATIC MEAN ANTRO POSTERIOR STABILITY INDEX UNILATERAL UNINVOLVED STANCE EYES CLOSE

SMLOSIUSUEO=STATIC MEAN MEDIO LATERAL STABILITY INDEX UNILATERALUN INVOLVED STANCE EYES OPEN

SMLOSIUSUEC=STATIC MEAN MEDIO LATERAL STABILITY INDEX UNILATERAL UNINVOLVED STANCE EYES CLOSE

**Table 5:** Mean±SD Dynamic stability indices in control group

	First day (Mean±SD)	After fifty weeks (Mean±SD)	p-value
**DMOSIBEO**	1.7±0.64	1.7±0.64	0.99
**DMOSIBEC**	7.6±3.3	7.6±3.3	0.99
**DMAPSIBEO**	1.81±2.76	1.81±2.76	0.99
**DMAPSIBEC**	5.08±1.86	5.08±1.86	0.99
**DMMLSIBEO**	1.64±.2.38	1.64±.2.38	0.99
**DMMLSIBEC**	4.53±2.61	4.89±2.58	0.34
**DMOSIUSIEO**	6.57±14.33	2.4±1.53	0.34
**DMOSIUSIEC**	4.8±2.44	4.8±2.44	0.99
**DMAPSIUSIEO**	1.98±1.94	1.98±1.94	0.99
**DMAPSIUSIEC**	3.45±2.05	3.45±2.05	0.99
**DMMLSIUSIEO**	1.66±1.38	1.66±1.38	0.99
**DMMLSIUSIEC**	2.68±1.29	2.68±1.29	0.99
**DMOSIUSUEO**	2.2±0.7	2.2±0.7	0.99
**DMOSIUSUEC**	5.15±2.69	5.15±2.69	0.99
**DMAPSIUSUEO**	1.75±1.39	1.75±1.39	0.99
**DMAPSIUSUEC**	4.89±2.39	4.89±2.39	0.99
**DMMLSIUSUEO**	1.55±1.01	1.55±1.01	0.99
**DMMLSIUSUEC**	2.54±1.34	5.03±8.28	0.34

* Significant P<0.05.

DMOSIBEO=DYNAMIC MEAN OVERALL STABILITY INDEX BILATERAL STANCE EYES OPEN

DMOSIBEC=DYNAMIC MEAN OVERALL STABILITY INDEX BILATERAL STANCE EYES CLOSE

DAPOSIBEO=DYNAMIC MEAN ANTRO POSTERIOR STABILITY INDEX BILATERAL STANCE EYES OPEN

DAPOSIBEC=DYNAMIC MEAN ANTRO POSTERIOR STABILITY INDEXBILATERAL STANCE EYES CLOSE

DMMLOSIBEO=DYNAMIC MEAN MEDIO LATERAL STABILITY INDEX BILATERAL STANCE EYES OPEN

DMMLOSIBEC=DYNAMIC MEAN MEDIO LATERAL STABILITY INDEX BILATERAL STANCE EYES CLOSE

DMOSIUSIEO=DYNAMIC MEAN OVERALL STABILITY INDEX UNILATERAL INVOLVED STANCE EYES OPEN

DMOSIUSIEC=DYNAMIC MEAN OVERALL STABILITY INDEX UNILATERAL INVOLVED STANCE EYES CLOSE

DAPOSIUSIEO=DYNAMIC MEAN ANTRO POSTERIOR STABILITY INDEX UNILATERAL INVOLVED STANCE EYES OPEN

DAPOSIUSIEC=DYNAMIC MEAN ANTRO POSTERIOR STABILITY INDEX UNILATERAL INVOLVED STANCE EYES CLOSE

DMLOSIUSIEO=DYNAMIC MEAN MEDIO LATERAL STABILITY INDEX UNILATERAL INVOLVED STANCE EYES OPEN

DMLOSIUSIEC=DYNAMIC MEAN MEDIO LATERAL STABILITY INDEX UNILATERAL INVOLVED STANCE EYES CLOSE

DMOSIUSUEO=DYNAMIC MEAN OVERALL STABILITY INDEX UNILATERAL UNINVOLVED STANCE EYES OPEN

DMOSIUSUEC=DYNAMIC MEAN OVERALL STABILITY INDEX UNILATERAL UN INVOLVED STANCE EYES CLOSE

DAPOSIUSUEO=DYNAMIC MEAN ANTRO POSTERIOR STABILITY INDEX UNILATERALUN INVOLVED STANCE EYES OPEN

DAPOSIUSUEC=DYNAMIC MEAN ANTRO POSTERIOR STABILITY INDEX UNILATERAL UNINVOLVED STANCE EYES CLOSE

DMLOSIUSUEO=DYNAMIC MEAN MEDIO LATERAL STABILITY INDEX UNILATERALUN INVOLVED STANCE EYES OPEN

DMLOSIUSUEC=DYNAMIC MEAN MEDIO LATERAL STABILITY INDEX UNILATERAL UNINVOLVED STANCE EYES

Independent t-test was used to compare static and dynamic stability indices between two groups. Tables 6-7 shows that there was not significant differences between both groups (p>0.05). Although amount of dynamic stability indices were decreased in balance training group, there were not significant differences between groups (p>0.05).

**Table 6:** Mean±SD static stability indices in control and balance training group

	Control Group (Mean±SD)	Balance Training Group (Mean±SD)	p-value
**SMOSIBEO**	0.46±0.23	0.39±0.26	0.52
**SMOSIBEC**	1.84±0.38	1.28±0.62	0.67
**SMAPSIBEO**	0.38±0.23	0.32±0.21	0.52
**SMAPSIBEC**	0.98±0.42	0.99±0.53	0.95
**SMMLSIBEO**	1.84±0.72	0.17±0.14	0.72
**SMMLSIBEC**	0.49±0.27	0.52±0.43	0.82
**SMOSIUSIEO**	1.25±0.46	1.03±0.64	0.39
**SMOSIUSIEC**	2.83±1.52	3.02±1.08	0.75
**SMAPSIUSIEO**	0.9±0.38	0.73±0.5	0.39
**SMAPSIUSIEC**	2.1±1.33	2.58±1.72	0.41
**SMMLSIUSIEO**	0.8±0.62	0.59±0.26	0.39
**SMMLSIUSIEC**	1.45±0.87	1.16±0.34	0.36
**SMOSIUSUEO**	1.18±0.83	1.05±0.65	0.7
**SMOSIUSUEC**	2.83±1.33	2.45±0.63	0.47
**SMAPSIUSUEO**	0.83±0.54	0.85±0.16	0.94
**SMAPSIUSUEC**	2.33±1.07	2.63±1.39	0.28
**SMMLSIUSUEO**	0.62±0.67	0.45±0.23	0.47
**SMMLSIUSUEC**	1.35±0.37	1.23±0.42	0.51

* Significant P<0.05.

SMOSIBEO=STATIC MEAN OVERALL STABILITY INDEX BILATERAL STANCE EYES OPEN

SMOSIBEC=STATIC MEAN OVERALL STABILITY INDEX BILATERAL STANCE EYES CLOSE

SAPOSIBEO=STATIC MEAN ANTRO POSTERIOR STABILITY INDEX BILATERAL STANCE EYES OPEN

SAPOSIBEC=STATIC MEAN ANTRO POSTERIOR STABILITY INDEX BILATERAL STANCE EYES CLOSE

SMMLOSIBEO=STATIC MEAN MEDIO LATERAL STABILITY INDEX BILATERAL STANCE EYES OPEN

SMMLOSIBEC=STATIC MEAN MEDIO LATERAL STABILITY INDEX BILATERAL STANCE EYES CLOSE

SMOSIUSIEO=STATIC MEAN OVERALL STABILITY INDEX UNILATERAL INVOLVED STANCE EYES OPEN

SMOSIUSIEC=STATIC MEAN OVERALL STABILITY INDEX UNILATERAL INVOLVED STANCE EYES CLOSE

SAPOSIUSIEO=STATIC MEAN ANTRO POSTERIOR STABILITY INDEX UNILATERAL INVOLVED STANCE EYES OPEN

SAPOSIUSIEC=STATIC MEAN ANTRO POSTERIOR STABILITY INDEX UNILATERAL INVOLVED STANCE EYES CLOSE

SMLOSIUSIEO=STATIC MEAN MEDIO LATERAL STABILITY INDEX UNILATERAL INVOLVED STANCE EYES OPEN

SMLOSIUSIEC=STATIC MEAN MEDIO LATERAL STABILITY INDEX UNILATERAL INVOLVED STANCE EYES CLOSE

SMOSIUSUEO=STATIC MEAN OVERALL STABILITY INDEX UNILATERAL UNINVOLVED STANCE EYES OPEN

SMOSIUSUEC=STATIC MEAN OVERALL STABILITY INDEX UNILATERAL UN INVOLVED STANCE EYES CLOSE

SAPOSIUSUEO=STATIC MEAN ANTRO POSTERIOR STABILITY INDEX UNILATERALUN INVOLVED STANCE EYES OPEN

SAPOSIUSUEC=STATIC MEAN ANTRO POSTERIOR STABILITY INDEX UNILATERAL UNINVOLVED STANCE EYES CLOSE

SMLOSIUSUEO=STATIC MEAN MEDIO LATERAL STABILITY INDEX UNILATERALUN INVOLVED STANCE EYES OPEN

SMLOSIUSUEC=STATIC MEAN MEDIO LATERAL STABILITY INDEX UNILATERAL UNINVOLVED STANCE EYES CLOSE

**Table 7:** Mean±SD dynamic stability indices in control and balance training group

	Control Group (Mean±SD)	Balance Training Group (Mean±SD)	p-value
**DMOSIBEO**	1.7±0.64	1.67±0.77	0.92
**DMOSIBEC**	7.6±3.3	5.16±3.16	0.11
**DMAPSIBEO**	1.81±2.76	1.12±0.58	0.47
**DMAPSIBEC**	5.08±1.86	4.03±2.58	0.29
**DMMLSIBEO**	1.64±2.38	0.96±0.41	0.42
**DMMLSIBEC**	4.89±2.58	2.53±1.44	0.05
**DMOSIUSIEO**	2.4±1.53	1.87±0.74	0.35
**DMOSIUSIEC**	4.8±2.44	4.06±1.9	0.46
**DMAPSIUSIEO**	1.98±1.94	1.15±0.36	0.23
**DMAPSIUSIEC**	3.45±2.05	2.94±1.69	0.56
**DMMLSIUSIEO**	1.66±1.38	1.19±67	0.36
**DMMLSIUSIEC**	2.68±1.29	2.07±0.86	0.24
**DMOSIUSUEO**	2.2±0.7	1.86±0.94	0.35
**DMOSIUSUEC**	5.15±2.69	3.87±1.49	0.19
**DMAPSIUSUEO**	1.75±1.39	1.16±0.63	0.26
**DMAPSIUSUEC**	4.89±2.39	3.07±1.33	0.55
**DMMLSIUSUEO**	1.55±1.01	1.11±0.73	0.32
**DMMLSIUSUEC**	5.03±8.28	1.74±0.55	0.26

* Significant P<0.05.

DMOSIBEO=DYNAMIC MEAN OVERALL STABILITY INDEX BILATERAL STANCE EYES OPEN

DMOSIBEC=DYNAMIC MEAN OVERALL STABILITY INDEX BILATERAL STANCE EYES CLOSE

DAPOSIBEO=DYNAMIC MEAN ANTRO POSTERIOR STABILITY INDEX BILATERAL STANCE EYES OPEN

DAPOSIBEC=DYNAMIC MEAN ANTRO POSTERIOR STABILITY INDEXBILATERAL STANCE EYES CLOSE

DMMLOSIBEO=DYNAMIC MEAN MEDIO LATERAL STABILITY INDEX BILATERAL STANCE EYES OPEN

DMMLOSIBEC=DYNAMIC MEAN MEDIO LATERAL STABILITY INDEX BILATERAL STANCE EYES CLOSE

DMOSIUSIEO=DYNAMIC MEAN OVERALL STABILITY INDEX UNILATERAL INVOLVED STANCE EYES OPEN

DMOSIUSIEC=DYNAMIC MEAN OVERALL STABILITY INDEX UNILATERAL INVOLVED STANCE EYES CLOSE

DAPOSIUSIEO=DYNAMIC MEAN ANTRO POSTERIOR STABILITY INDEX UNILATERAL INVOLVED STANCE EYES OPEN

DAPOSIUSIEC=DYNAMIC MEAN ANTRO POSTERIOR STABILITY INDEX UNILATERAL INVOLVED STANCE EYES CLOSE

DMLOSIUSIEO=DYNAMIC MEAN MEDIO LATERAL STABILITY INDEX UNILATERAL INVOLVED STANCE EYES OPEN

DMLOSIUSIEC=DYNAMIC MEAN MEDIO LATERAL STABILITY INDEX UNILATERAL INVOLVED STANCE EYES CLOSE

DMOSIUSUEO=DYNAMIC MEAN OVERALL STABILITY INDEX UNILATERAL UNINVOLVED STANCE EYES OPEN

DMOSIUSUEC=DYNAMIC MEAN OVERALL STABILITY INDEX UNILATERAL UN INVOLVED STANCE EYES CLOSE

DAPOSIUSUEO=DYNAMIC MEAN ANTRO POSTERIOR STABILITY INDEX UNILATERALUN INVOLVED STANCE EYES OPEN

DAPOSIUSUEC=DYNAMIC MEAN ANTRO POSTERIOR STABILITY INDEX UNILATERAL UNINVOLVED STANCE EYES CLOSE

DMLOSIUSUEO=DYNAMIC MEAN MEDIO LATERAL STABILITY INDEX UNILATERALUN INVOLVED STANCE EYES OPEN

DMLOSIUSUEC=DYNAMIC MEAN MEDIO LATERAL STABILITY INDEX UNILATERAL UNINVOLVED STANCE EYES

## 4. Discussion

These results support that balance exercise could decrease dynamic stability indices in subjects with ACL reconstruction. In this study, static and dynamic stability indices, however, did not significantly changed in ACL reconstruction subjects after balance training. The result of this experimental study suggests that proprioceptive and balance exercises improve postural stability in subjects with ACL deficiency in early stage of ACL reconstruction rehabilitation. These findings, also, suggest that neuromuscular training could commonly utilized for patients with ACL reconstruction in clinical management.

It is speculate that proprioceptive and balance training might enhance neuromotor recruitment, thus enhancing muscle strength (Copper et al., 2005). Based on this hypothesis, many studies have been performed (Ageberg et al., 2001; Ageberg, 2002; Liu-Ambrose et al., 2003; Beard et al., 1994; Fitzgerald et al., 2000; Zatterstrom et al., 2000). In several studies provided that proprioceptive and balance exercises improve functional outcomes in people with ACL-deficient knees (Ageberg et al., 2001; Ageberg, 2002; Liu-Ambrose et al., 2003; Beard et al., 1994). In addition, many evidences confirmed in Improvements of joint position sense, muscle strength, perceived knee joint function, and hop testing following proprioceptive and balance exercises (Ageberg et al., 2001; Ageberg, 2002; Liu-Ambrose et al., 2003; Beard et al., 1994; Fitzgerald et al., 2000; Zatterstrom et al., 2000). Furthermore, some evidences supported no detrimental effects such as increased passive joint laxity or decrease in strength (Ageberg et al., 2001; Ageberg, 2002; Liu-Ambrose et al., 2003; Beard et al., 1994).

It should be noted that there were many diversity of outcomes in several ACL reconstruction studies (Cooper et al., 2005; Pizzari et al., 2005; Lui-Ambrose et al., 2003). Cooper et al., (2005) in one of the randomized clinical trial to investigate proprioceptive and balance training following ACL reconstruction, and the first trial to investigate this in the early phases of rehabilitation (Cooper et al., 2005). They showed that there was either no difference between the two forms of exercise (Balance exercise and strengthening exercise) or strength training may be more beneficial than proprioceptive and balance training in the early phase of rehabilitation after ACL reconstructive surgery. Cooper et al., (2005) measured hop test and several clinical tests. Any significant changes were not seen in clinical tests and functional outcomes following 6 weeks balance training (Cooper et al., 2005). Therefore, they proposed that there was no advantage in proprioceptive and balance exercises in the early phases of rehabilitation following ACL reconstruction when compared with a traditional strengthening program (Cooper et al., 2005). Vickers and Altman (2001) confirmed that it may be the case that strength training is the best type of training in the early phases of rehabilitation following ACL reconstruction (Vickers and Altman 2001). In our study, static and dynamic postural stability indices were measured by Biodex system. Our result was similar to those of Cooper et al., (2005), Vickers and Altman (2001) studies. Current results showed that static and dynamic postural stability indices did not significantly change after 2 weeks balance exercises in early stage of ACL rehabilitation. In addition, it is reported that better outcomes after ACL reconstructive surgery have been observed in participants under the age of 30 years (Pizzari et al., 2005). Liu-Ambrose et al., (2003) compared proprioceptive and balance training with strength training following ACL reconstruction (Liu-Ambrose et al., 2003). They investigated 10 participants over a 12-week training program. The results of their study were similar to those of Cooper et al., (2005). Liu-Ambrose et al., (2003) appeared to be no additional benefit in performing proprioceptive and balance training following ACL reconstruction with regards to patient functional activity (Liu-Ambrose et al., 2003). It should be noted that in our study and Cooper et al., (2005) study, the intervention was carried out in the early phases of rehabilitation, while Liu-Ambrose et al., (2003) study investigated the effects of balance and proprioceptive exercises in the later phases of rehabilitation. In dissimilarity result, Risberg et al., (2007) indicated that, although there were small differences between the neuromuscular program and the strengthening program, the neuromuscular program was superior to the strengthening program in improving knee function after ACL reconstruction. The Cincinnati Knee Score and VAS was significantly better after 6 months of the neuromuscular program compared with 6 months of the strengthening program (Risberg et al., 2007). It seems that lack of common protocol in neuromuscular training, variety of assessment, duration of training and onset of rehabilitation program lead to different results or outcome following ACL reconstruction rehabilitation.

For the other causal factors in postural stability, studies suggested that quadriceps weakness was common in the ACL-reconstructed leg (Kobayashi et al., 2004, Marcacci et al., 1998, Keays et al., 2001, Konishi et al., 2002). Reider et al. (2003) also investigated the correlation between strength and proprioception and found that there was a positive correlation between proprioception and quadriceps strength. However, in our study, strength of quadriceps and hamstring and H/Q ratio were not evaluated. Konishi et al. (2002) suggested that restoration of the sensory function of ACL was necessary to improve muscle strength, based on the abnormal γ-loop of vastus lateralis and vastus medialis muscles after ACL reconstruction (Konishi et al., 2002). Another study showed that strength training alone promoted proprioception (Bouët & Gahéry 2000). However, although Reider et al. (2003) found a positive correlation existed between the muscle strength and proprioception, they could not conclude which of the two plays the active role in the causality. The positive correlation between proprioception and muscle strength suggests that strength training and proprioception training could be mutually beneficial (Zhou et al., 2008).

There still remain other factors which may affect proprioceptive recovery, such as age, pain, methods of surgical operation, rehabilitation training, and recovering duration (Aydoğ et al., 2006; Bennell et al., 2005). Also, several study reported gender and H/Q ratio no influence on proprioception (Zhou et al., 2008). It is well known that females have a greater risk of ACL rupture compared with males, and studies have attributed this increased risk to differences in anatomic, hormonal, and material properties and movement patterns (Chandrashekar et al, 2006; Schmitz et al., 2007; Borotikar et al., 2008). In contrast, there is no evidence to suggest a proprioceptive difference between males and females (Zhou et al., 2008).

Variety in study's results and different factors which effect ACL neuromuscular training program noted that long-term follow-up is needed to determine whether this modest difference in neuromuscular exercises has any long-term consequences. Further research also is needed to disclose possible mechanisms for these different exercises.

## 5. Conclusion

In summary, our findings demonstrate that balance exercise could decrease dynamic stability indices in subjects with ACL reconstruction. Proprioceptive and balance exercise improved postural stability in subjects with ACL deficiency in early stage of ACL reconstruction rehabilitation. These findings, also, suggest that neuromuscular training could commonly utilized for patients with ACL reconstruction in clinical management.

## References

[ref1] Ageberg E, Zatterstrom R, Moritz U, Friden T (2001). Influence of supervised and nonsupervised training on postural control after an acute anterior cruciate ligament rupture: A three-year longitudinal prospective study. Journal of Orthopaedic and Sports Physical Therapy.

[ref2] Ageberg E (2002). Consequences of a ligament injury on neuromuscular function and relevance to rehabilitation –using the anterior cruciate ligament-injured knee as model. J Electromyogr Kinesiol.

[ref3] Akbari A, Asiaei F, Farahani A (2014a). Comparison of the Effect of Static Stretching, Exercises and Vibration on Postural Stability Indices in Healthy Women. J Rafsanjan Univ Med Sci.

[ref4] Akbari A, Sarmadi A, Zafardanesh P (2014b). The Effect of Ankle, Taping and Balance Exercises on Postural Stability Indices in Healthy Women. J Phys Ther Sci.

[ref5] Aydoğ S. T, Korkusuz P, Doral M. N, Tetik O, Demirel H. A (2006). Decrease in the numbers of mechanoreceptors in rabbit ACL: the effects of ageing. Knee Surg Sports Traumatol Arthrosc.

[ref6] Barrack R. L, Skinner H. B, Buckley S. L (1989). Proprioception in the anterior cruciate deficient knee. American Journal of Sports Medicine.

[ref7] Barrett D. S (1991). Proprioception and function after anterior cruciate reconstruction. Journal of Bone and Joint Surgery, Series.

[ref8] Beard D. J, Dodd C. A, Trundle H. R, Simpson A. H (1994). Proprioception enhancement for anterior cruciate ligament deficiency: A prospective randomised trial of two physiotherapy regimes. Journal of Bone & Joint Surgery—British.

[ref9] Bennell K, Wee E, Crossley K, Stillman B, Hodges P (2005). Effects of experimentally-induced anterior knee pain on knee joint position sense in healthy individuals. J Orthop Res.

[ref10] (1999). Biodex stability system, Instruction manual system. Biodex medical systems.

[ref11] Borotikar B. S, Newcomer R, Koppes R, McLean S. G (2008). Combined effects of fatigue and decision making on female lower limb landing postures: central and peripheral contributions to ACL injury risk. Clin Biomech (Bristol, Avon).

[ref12] Bouët V, Gahéry Y (2000). Muscular exercise improves knee position sense in humans. Neurosci Lett.

[ref13] Brukner P. D, Khan K (2001). Clinical Sports Medicine.

[ref14] Chandrashekar N, Mansouri H, Slauterbeck J, Hashemi J (2006). Sex-based differences in the tensile properties of the human anterior cruciate ligament. J Biomech.

[ref15] Chmielewski T. L, Rudolph K. S, Snyder-Mackler L (2002). Development of dynamic knee stability after acute ACL injury. J Electromyogr Kinesiol.

[ref16] Cooper R. L, Taylor N. F, Feller J. A (2005). A Randomised Controlled Trial of Proprioceptive and Balance Training after Surgical Reconstruction of The Anterior Cruciate Ligament. Research in Sports Medicine.

[ref17] Cooper R. L, Taylor N. F, Feller J. A (2005). A Systematic Review of the Effect of Proprioceptive and Balance Exercises on People With an Injured or Reconstructed Anterior Cruciate Ligament. Research in Sports Medicine: An International Journal.

[ref18] Corrigan J. P, Cashman W. F, Brady M. P (1992). Proprioception in the cruciate deficient knee. Journal of Bone and Joint Surgery–Series B.

[ref19] Fitzgerald G. K, Axe M. J, Snyder Mackler L (2000). The efficacy of perturbation training in nonoperative anterior cruciate ligament rehabilitation programs for physically active individuals. Physical Therapy.

[ref20] Johansson H, Sjolander P, Sojka P (1991). A sensory role for the cruciate ligaments. Clinical Orthopaedics & Related Research.

[ref21] Keays S. L, Bullock-Saxtona J, Keays A. C, Newcombe P (2001). Muscle strength and function before and after anterior cruciate ligament reconstruction using semitendonosus and gracilis. Knee.

[ref22] Kobayashi A, Higuchi H, Terauchi M, Kobayashi F, Kimura M, Takagishi K (2004). Muscle performance after anterior cruciate ligament reconstruction. Int Orthop.

[ref23] Konishi Y, Fukubayashi T, Takeshita D (2002). Mechanism of quadriceps femoris muscle weakness in patients with anterior cruciate ligament reconstruction. Scand J Med Sci Sports.

[ref24] Lewek M, Rudolph K. S, Axe M. J, Snyder-Mackler L (2002). The effect of insufficient quadriceps strength on gait after anterior cruciate ligament reconstruction. Clin Biomech (Bristol, Avon).

[ref25] Liu-Ambrose T, Taunton J. E, MacIntyre D, McConkey P, Khan K. M (2003). The effects of proprioceptive or strength training on the neuromsucular function of the ACL reconstructed knee: a randomized clinical trial. Scandinavian Journal of Medicine & Science in Sports.

[ref26] Marcacci M, Zaffagnini S, Iacono F, Neri M.P, Loreti I, Petitto A (1998). Arthroscopic intra- and extra-articular anterior cruciate ligament reconstruction with gracilis and semitendinosus tendons. Knee Surg Sports Traumatol Arthrosc.

[ref27] Mou-wang Z, Li G, Ya-ping C, Chang-long Y, Ying-fang A, Hong-shi H, Yan-yan Y (2008). Factors affecting proprioceptive recovery after anterior cruciate ligament reconstruction. Chin Med J.

[ref28] Pizzari T, Taylor N. F, McBurney H, Feller J. A (2005). Adherence to rehabilitation following anterior cruciate ligament reconstructive surgery: Implications for outcome. Journal of Sport Rehabilitation.

[ref29] Reider B, Arcand M. A, Diehl L.H, Mroczek K, Abulencia A, Stroud C. C (2003). Proprioception of the knee before and after anterior cruciate ligament reconstruction. Arthroscopy.

[ref30] Risberg M. A, Holm I, Myklebust G, Engebretsen L (2007). Neuromuscular Training Versus Strength Training During First 6 Months After Anterior Cruciate Ligament Reconstruction: A Randomized Clinical Trial. Phys Ther.

[ref31] Risberg M. S, Lewek M, Snyder-Mackler L (2004). A systematic review of evidence for anterior cruciate ligament rehabiltation: How much and what type?. Physical Therapy in Sport.

[ref32] Rudolph K. S, Eastlack M. E, Axe M. J, Snyder-Mackler L (1998). Basmajian Student Award Paper: Movement patterns after anterior cruciate ligament injury: a comparison of patients who compensate well for the injury and those who require operative stabilization. J Electromyogr Kinesiol.

[ref33] Schmitz R. J, Kulas A. S, Perrin D. H, Riemann B. L, Shultz S. J (2007). Sex differences in lower extremity biomechanics during single leg landings. Clin Biomech (Bristol, Avon).

[ref34] Snyder-Mackler L, Fitzgerald G. K, Bartolozzi A. R, Ciccotti M. G (1997). The relationship between passive joint laxity and functional outcome after anterior cruciate ligament injury. Am J Sports Med.

[ref35] Swanik C. B, Lephart S. M, Giannantonio F. P, Fu F. H (1997). Reestablishing proprioception and neuromuscular control in the ACL-injured athlete. Journal of Sport Rehabilitation.

[ref36] Vickers A. J, Altman D. G (2001). Analysing controlled trials with baseline and follow up measurements. British Medical Journal.

[ref37] Zatterstrom R, Friden T, Lindstrand A, Moritz U (2000). Rehabilitation following acute anterior cruciate ligament injuries—A 12-month follow-up of a randomized clinical trial. Scandinavian Journal of Medicine & Science in Sports.

